# Anchoring Bias, Lyme Disease, and the Diagnosis Conundrum

**DOI:** 10.7759/cureus.4300

**Published:** 2019-03-22

**Authors:** Luis E Aguirre, Teresa Chueng, Marco Lorio, Michael Mueller

**Affiliations:** 1 Internal Medicine, University of Miami Miller School of Medicine/Jackson Memorial Hospital, Miami, USA

**Keywords:** anchoring bias, lyme disease, tickborne diseases, ticks, borrelia, immunoblot, false positive

## Abstract

Lyme disease remains the most common vector-borne disease in North America. This academic teaching case highlights a full diagnostic workup fueled by anchoring bias, resulting in a presumptive diagnosis of early disseminated Lyme meningitis. Patient report of direct tick exposure, neurocranial defects, and equivocal serologies, despite geographic region of low pretest probability, confounded the clinical picture. Infectious workup confirmed the true diagnosis to be aseptic meningitis due to enterovirus. This clinical vignette acknowledges the habitual anchoring biases in the daily decision-making among internists and trainees contributing to misdiagnoses and subsequently, overtreatment.

## Introduction

Lyme disease, a borreliosis precipitated by the infected Ixodes scapularis tick, remains the most common vector-borne disease in North America [1]. Transmission of the spirochete causes febrile illness alongside multiorgan complications of meningitis, arthritis, and facial palsy. Lyme disease in Florida remains rare but underreported, with 0.6 cases per 100,000 inhabitants yearly [2]. Of 216 regional cases reported in 2016, 132 were confirmed positive by the CDC [2]. Physicians and trainees habitually grapple with cognitive biases during a workup which can lead to anchoring and perpetuate overtreatment. With a priori knowledge of high IgM false seropositivity to Lyme and history of exposure to ticks, should the clinical picture take precedence over the known distribution of disease?

## Case presentation

A 29-year-old male presented for two weeks of excruciating holocranial cephalgia accompanied by fever, myalgia, and diarrhea. He developed facial paresis with nonfocal paresthesia, bilateral scotomas, and a self-resolved erythematous patch along his inner thigh weeks prior. He endorsed dog ticks at home in Miami, Florida, but denied bites. Visual fields showed inferotemporal compromise bilaterally. Left gaze was restricted by horizontal binocular diplopia. Cranial nerves were otherwise intact and the remainder of the neurological exam was unremarkable, though he was incapable of sustaining right handgrip. Western blot demonstrated positive IgM and negative IgG for *Borrelia burgdorferi*. Electrocardiogram was negative. Lumbar puncture revealed clear cerebrospinal fluid (CSF) of 84 white blood cells, 96% lymphocytes, and 110 protein. Despite an atypical geographical context, he received a presumptive diagnosis of early disseminated Lyme meningitis that was treated empirically with doxycycline. Subsequent CSF polymerase chain reaction was negative for *B. burgdorferi*, *B. lonestari*, and tick-borne encephalitis. The viral panel was positive for Echovirus 30 and Coxsackie B5. His headache and vision improved gradually; however, the patient experienced distress from misdiagnosis with a life-threatening and contagious illness affecting family contact and financial burden from prolonged work leave.

## Discussion

**Figure 1:**
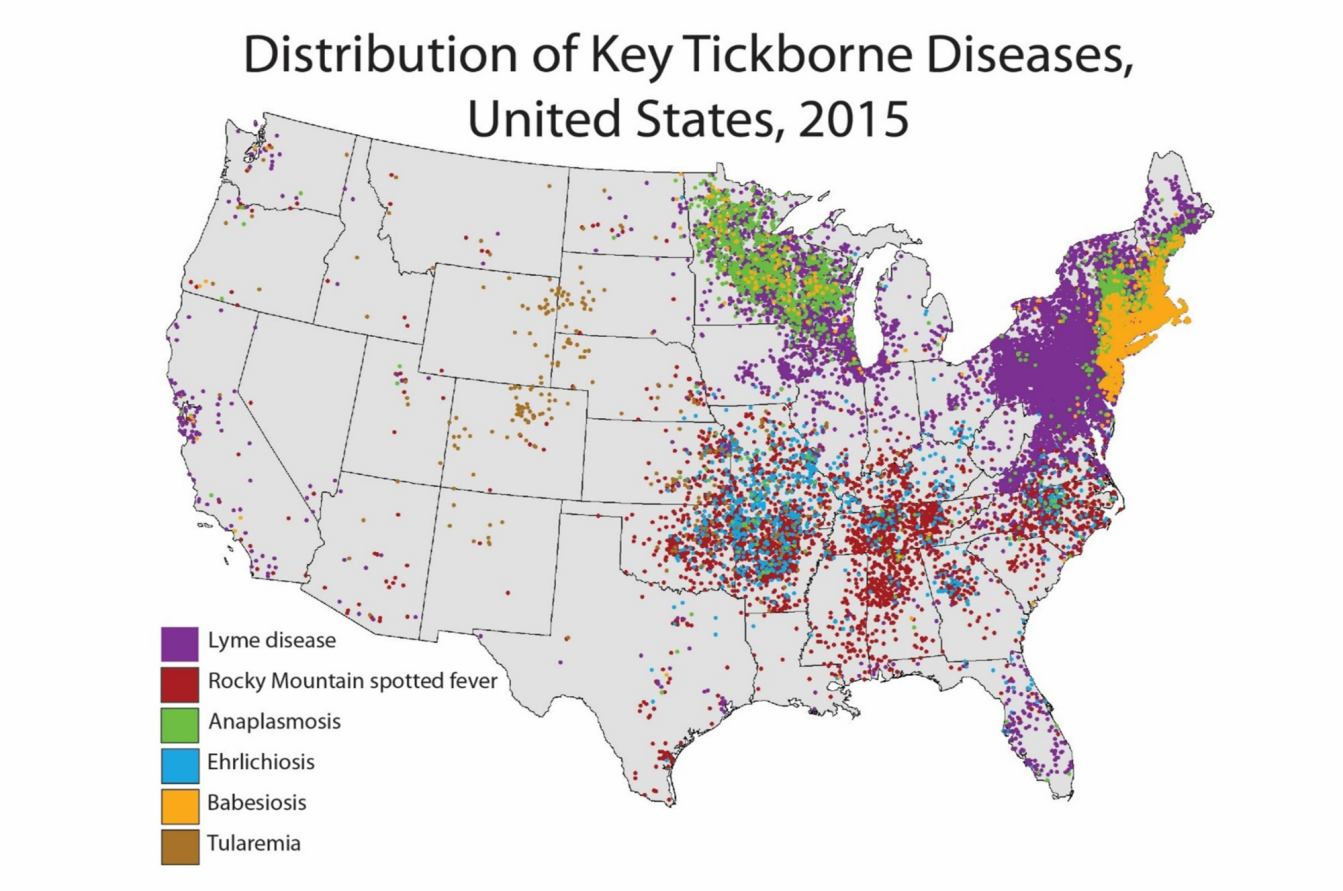
Distribution of key tick-borne diseases, United States, 2015 Each dot represents one case. Cases are reported from the infected person’s county of residence, not necessarily the place where they were infected.

Lyme disease (LD), a borreliosis with potential to cause febrile illness alongside multiorgan complications of meningoencephalitis, migratory rash with central clearing, carditis, facial palsy, and arthritis after inoculation of a spirochete through the tick bite, is the most common vector-borne disease in North America [1]. Endemicity spans the north and central Atlantic coast, as well as focal regions of the Pacific (Figure 1). Precipitated by the infected Ixodes scapularis tick, Lyme disease in Florida is rare but underreported, with 0.6 cases per 100,000 inhabitants yearly [2].

Physicians and trainees habitually grapple with cognitive biases during a workup which can lead to anchoring and perpetuate overtreatment. In this narrative, anchoring bias of the more unusual Lyme meningoencephalitis, given the history of tick exposure and neurological complications with rash led to the overlooking of the more common echovirus/coxsackie infection likely to cause symptoms. While doxycycline was the correct empiric treatment for Lyme meningitis, patient psychological and financial distress would have been preventable had anchoring bias been acknowledged and the CDC two-tiered testing approach was used during work-up.

**Figure 2:**
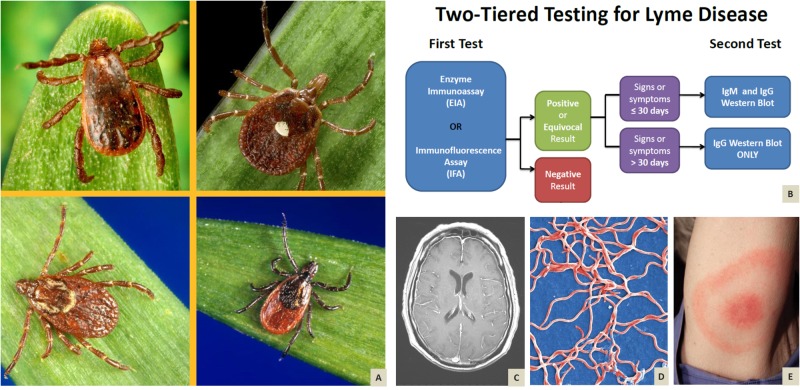
Tick montage and characteristic features of Lyme disease (A) Clockwise from the upper left: Female American dog tick, *Dermacentor variabilis*, vector for Rocky Mountain spotted fever (RMSF), and tularemia. Dorsal view of a female “lone star tick”, *Amblyomma americanum*. *A. americanum *is found through the eastern and south-central states and transmits *Ehrlichia chaffeensis* and *Ehrlichia ewingii*. Dorsal view of a female black-legged tick, *Ixodes scapularis*, known to transmit *Borrelia burgdorferi*. Male brown dog tick, *Rhipicephalus sanguineus*, from a dorsal view. *R. sanguineus* has been found to be a less-common vector for Rocky Mountain spotted fever (RMSF). (B) Two-tiered testing decision tree for Lyme Disease. (C) Brain MRI T1-weighted image with contrast depicting meningeal enhancement. (D) Digitally colorized scanning electron microscopic image depicting a grouping of numerous, Gram-negative, anaerobic, *B. burgdorferi* bacteria, which had been derived from a pure culture. (E) Pathognomonic erythematous rash in the pattern of a “bull’s-eye”, manifesting at the site of a tick bite.

CDC guidelines encourage a two-tiered testing approach for diagnosing Lyme disease in patients meeting all of the following criteria: a) residing or with a recent history of travel to an endemic area, b) exposure to ticks, and c) symptoms consistent with either early disseminated or late Lyme disease [3]. Either EIA (enzyme immunoassay) or IFA (immunofluorescence assay) should be performed initially based on clinical suspicion. Positive or equivocal results must be followed by confirmatory western blot with specific serologies requested based on duration of disease: both IgM and IgG should be tested for in patients with signs and symptoms lasting less than 30 days, whereas sole assessment of IgG should be pursued in those with chronicity greater than a month (Figure 2B). 

Early disseminated disease usually presents with positivity to both IgM and IgG antibodies to *B. burgdorferi*. The IgM immunoblot is a valuable diagnostic tool when used in this context, given that IgG serologies require more time to result. Extemporal and noncontextual assessment can be detrimental given its poor positive predictive value in patients lacking cardinal features of Lyme disease. The high false positive rate of 27.5% associated with the IgM immunoblot is reported in one case series underpinning this assertion [4].

In a cross-sectional study by Lantos et al, 7289 serologic tests for Lyme were analyzed over a period of 7 years. Only 17% of specimens (n = 1216) were found to have a positive or equivocal EIA warranting immunoblot confirmation. Of that subset, 372 (30%) were dismissed as negative cases and 854 were confirmed positives (11.7% of all Lyme tests) [5]. Of the 167 cases identified as IgM(+) and IgG(-), 48 were reported as false positives (28.7%).

The financial burden that overdiagnosis and overtreatment of Lyme disease exert on society takes shape in the material cost arising from unnecessary testing, medication expenses and sick leaves leading to time lost away from work and school, not to mention the non-quantifiable emotional distress on the patient assuming the sick role and their caretakers [6].

## Conclusions

This case illustrates the consequences of anchor bias and the financial and emotional burden it carries in the context of Lyme disease. Initial suspicion for early disseminated Lyme with aseptic meningitis was fueled by tick exposure, cutaneous exanthem, and facial palsy. However, the two-tiered approach in diagnosing Lyme disease must be used, with high IgM false positivity in mind. The final diagnosis was aseptic meningitis due to enterovirus, given positive echovirus/coxsackie and preceding viral illness in a low pretest population for Lyme. Anchoring during workup can perpetuate misdiagnoses and cause harm. In approach to honing clinical judgment and decision-making, management should always take into context pretest probability, as well as social and environmental determinants of health.
